# Alterations of the bacterial ocular surface microbiome are found in both eyes of horses with unilateral ulcerative keratitis

**DOI:** 10.1371/journal.pone.0291028

**Published:** 2023-09-08

**Authors:** Martha E. Julien, Johnathan B. Shih, Bruna Correa Lopes, Lucien V. Vallone, Jan S. Suchodolski, Rachel Pilla, Erin M. Scott

**Affiliations:** 1 Department of Small Animal Clinical Sciences, School of Veterinary Medicine & Biomedical Sciences, Texas A&M University, College Station, Texas, United States of America; 2 Department of Clinical Sciences, College of Veterinary Medicine, Cornell University, Ithaca, New York, United States of America; Bayero University Kano, NIGERIA

## Abstract

Next generation sequencing (NGS) studies in healthy equine eyes have shown a more diverse ocular surface microbiota compared to culture-based techniques. This study aimed to compare the bacterial ocular surface microbiota in both eyes of horses with unilateral ulcerative keratitis (UK) with controls free of ocular disease. Conjunctival swabs were obtained from both ulcerated eyes and unaffected eyes of 15 client-owned horses with unilateral UK following informed consent, as well as from one eye of 15 healthy horses. Genomic DNA was extracted from the swabs and sequenced on an Illumina platform using primers that target the V4 region of bacterial 16S rRNA. Data were analyzed using Quantitative Insights Into Molecular Ecology (QIIME2). The ocular surface of ulcerated eyes had significantly decreased species richness compared with unaffected fellow eyes (Chao1 q = 0.045, Observed ASVs p = 0.045) with no differences in evenness of species (Shannon q = 0.135). Bacterial community structure was significantly different between either eye of horses with UK and controls (unweighted UniFrac: control vs. unaffected, p = 0.03; control vs. ulcerated, p = 0.003; unaffected vs. ulcerated, p = 0.016). Relative abundance of the gram-positive taxonomic class, Bacilli, was significantly increased in ulcerated eyes compared with controls (q = 0.004). Relative abundance of the taxonomic family Staphylococcaceae was significantly increased in ulcerated and unaffected eyes compared with controls (q = 0.030). The results suggest the occurrence of dysbiosis in infected eyes and reveal alterations in beta diversity and taxa of unaffected fellow eyes. Further investigations are necessary to better understand the role of the microbiome in the pathophysiology of ocular surface disease.

## Introduction

The use of next generation sequencing (NGS) to identify the ocular microbiota has allowed for a more-encompassing method of detection of microbial species in human medicine compared to previously described culture-based techniques [1]. This methodology can identify microbes that would previously remain undetected [2]. In recent years, 16S rRNA gene sequencing has become a viable option for identifying a diverse population of ocular microorganisms within veterinary species, such as cats [3, 4], horses [5–7], and dogs [8–11]. This modality within veterinary medicine has similarly proven advantageous in revealing bacterial microorganisms that do not grow well in laboratory conditions and would otherwise go unidentified with culture-based methods.

A previous study examined the bacterial ocular surface microbiome utilizing next generation sequencing (NGS) in clinically normal horses before and after treatment with topical neomycin-polymyxin-bacitracin and found at baseline, the most abundant phyla identified were Proteobacteria (46.1%), Firmicutes (24.6%), Actinobacteria (12.6%), and Bacteroidetes (11.2%). The most abundant families included Pasteurellaceae (13.7%), Sphingomonadaceae (7.9%), an unclassified Order of Cardiobacteriales (7.7%), and Moraxellaceae (4.8%) [5]. These findings added to the traditional microorganisms identified with culture-based techniques, with Gram-positive bacteria historically reported to predominate the equine ocular surface, including *Bacillus*, *Staphylococcus*, *Streptococcus*, and *Corynebacterium spp*., which have been commonly cultured regardless of geography, climate, or season [12–19]. As well, these findings contradicted the common belief that gram-positive microorganisms dominate the ocular surface. Furthermore, these findings show that that the major bacterial taxa on the equine ocular surface remain stable over time and following topical antibiotic therapy.

The equine ocular surface is at risk of developing blinding ocular disease such as ulcerative keratitis (UK) [18–26], which can be complicated by secondary bacterial and fungal infections requiring specific medical therapy tailored to the microorganisms present, or in some cases, surgical intervention. The resident ocular surface microbiota serves to protect and prevent the proliferation of pathogenic species and shifts in the homeostatic microbiome may be linked to infectious pathologies [1, 27, 28], as well external environmental factors. Implicating bacterial microorganisms in the development of ulcerative keratitis diagnosed with culture-based techniques include *Streptococcus spp*., *Staphylococcus spp*., and *Pseudomonas aeruginosa* [18–26].

In human medicine, an analysis of the microbial composition of healthy eyes and eyes with bacterial keratitis utilizing molecular sequencing found homeostatic ocular microorganisms to be sparse and the presence of a pathological microbiome dominated by *Pseudomonas aeruginosa* [27, 28].

The goal of this study was to determine the microbiota of horses with UK. Furthermore, to compare the microbiome of these eyes to the fellow eye devoid of disease and to the eyes of healthy horses serving as a control. Host factors were suspected to lead to changes in the ocular microbial community.

## Materials & methods

### Participants

The study was approved by the Texas A&M University Institutional Animal Care and Use Committee (Animal Use Protocols #2017–0333 and #2018–0237). Fifteen client-owned horses evaluated by the Comparative Ophthalmology Service with a diagnosis of UK in one eye and a healthy, unaffected fellow eye were enrolled in the study after owner consent was obtained. All cases had clinical signs suggestive of an infected corneal ulcer such as stromal loss, white blood cell infiltrate, varying degrees of keratomalacia, and reflex uveitis, although they still required a positive culture for a definitive diagnosis of infectious ulcerative keratitis. An additional 15 horses, free of ocular disease, were selected from the teaching herd at the Department of Large Animal Clinical Sciences at Texas A&M University School of Veterinary Medicine & Biomedical Sciences to serve as controls (Table 1). Randomization of sampled eyes from the healthy control group was determined using online software (https://www.randomizer.org).

**Table 1:** Control and study populations: Signalment and eyes sampled for analysis.

Study Population	Breed	Age (Y)	Sex	Ulcerated Eye	Unaffected Fellow Eye
**1**	Quarter Horse	9	MC	OS	OD
**2**	Quarter Horse	15	MC	OS	OD
**3**	Thoroughbred	10	F	OD	OS
**4**	Quarter Horse	7	MC	OS	OD
**5**	Quarter Horse	19	F	OS	OD
**6**	Quarter Horse	14	F	OD	OS
**7**	American Paint	9	MC	OD	OS
**8**	Quarter Horse	10	MC	OS	OD
**9**	Pony of Americas	6	F	OD	OS
**10**	Arabian	15	F	OD	OS
**11**	Quarter Horse	12	MC	OS	OD
**12**	Thoroughbred	2	F	OS	OD
**13**	Warmblood	2	F	OS	OD
**14**	American Miniature Horse	7	M	OS	OD
**15**	Thoroughbred	18	MC	OS	OD
**Control Population**	**Breed**	**Age (Y)**	**Sex**	**Sampled Eye**	
**1**	Quarter Horse	18	F	OS	
**2**	Quarter Horse	17	F	OD	
**3**	Quarter Horse	18	M	OD	
**4**	Quarter Horse-cross	23	M	OS	
**5**	Quarter Horse	18	M	OD	
**6**	Arabian	26	M	OD	
**7**	Quarter Horse	8	M	OD	
**8**	Quarter Horse	10	M	OS	
**9**	Quarter Horse	15	M	OS	
**10**	Quarter Horse	12	F	OD	
**11**	Quarter Horse-cross	15	F	OS	
**12**	Quarter Horse	10	F	OS	
**13**	Quarter Horse	20	F	OS	
**14**	Quarter Horse	24	F	OS	
**15**	Quarter Horse-cross	10	M	OD	

Abbreviations: Y: years, F: female (mare), M: male (stallion), MC: castrated male (gelding), OD: right eye, OS: left eye

#### Sample collection

A complete ophthalmic examination was performed on all horses by a board-certified veterinary ophthalmologist (EMS, LVV) and resident. The anterior segment of the eye was examined by slit-lamp biomicroscopy (SL-17, Kowa Optimed Inc., Torrance, CA), and the posterior segment of the eye by indirect ophthalmoscopy (Vantage Plus Wireless Headset, Keeler Instruments Inc., Malvern, PA), as previously described [29]. A routine minimal ophthalmic database consisted of fluorescein staining (Amcon Laboratories Inc., St. Louis, MO) and tonometry (Tono-Pen, Dan Scott and Associates, Inc., Westerville, OH).

Following the ophthalmic exam, conjunctival swab samples were collected for NGS sequencing. A volume of 0.2 ml 0.5% tetracaine (Bausch & Lomb Inc., Tampa, FL) was placed on the ocular surface of each eye to provide topical analgesia and allow for deep swabbing with applied pressure. The inferior conjunctival fornix of both the ulcerated eye and the fellow unaffected eye were sampled with Isohelix buccal swabs (Boca Scientific, Inc. Westwood, MA). Two swabs were used for each site, and each side of the swab was rubbed in the conjunctival fornix 10 times, as previously described [5, 29]. Samples were collected from the study population upon initial presentation to Texas A&M and prior to the application of antimicrobial therapy prescribed by the Comparative Ophthalmology Service. One eye from the healthy control horses was randomly selected and conjunctival swab samples were collected in the same manner and location for the control population. A volume of 0.2 ml 0.5% tetracaine was placed on two unused swabs immediately following the swabbing of healthy horses in the control population to serve as a negative sample control to rule out environmental contamination. The swabs were collected in DNeasy Powerbead tubes with 750-μl buffer containing guanidine thiocyanate (QIAGEN, Inc., Germantown, MD). All samples were stored at 4 degrees C until time for extractions.

Corneal cytology samples of eyes with UK were obtained from ulcerated corneal lesions following study sampling, with the blunt end of a 15-scalpel blade (Aspen Surgical^®^, Caledonia, MI) or a sterile cytology brush (Microbrush, Grafton, WI) and submitted to the Texas A&M University Veterinary Medical Teaching Hospital Clinical Pathology Laboratory and processed as described [30]. Aerobic bacterial and fungal culture samples were also obtained from eyes with UK by lightly swabbing ulcerated corneal lesions with a sterile transport swab (BD BBL CultureSwab Collection & Transport System, Becton Dickinson and Company, Franklin Lakes, NJ). Specimens were placed into sterile culturette tubes (BD BBL CultureSwab Collection & Transport System, Becton Dickinson and Company, Franklin Lakes, NJ) and immediately submitted to the Clinical Microbiology Laboratory at Texas A&M University. Specimens were processed within 24 hours of collection as previously described [31].

#### DNA extraction and sequencing

Genomic DNA was extracted from the conjunctival swabs and negative environmental control sample (unused swabs combined with 0.2 ml 0.5% tetracaine) with the DNeasy Powersoil DNA isolation kit (QIAGEN, Inc., Germantown, MD), based upon the manufacturer’s instructions.

Following DNA extraction, samples were shipped to a commercial laboratory for sequencing (MR DNA Laboratory, www.mrdna.com, Shallowater, TX, USA). Sequencing of the bacterial 16S rRNA gene V4 variable regions was performed using primers 515F (5’-GTGYCAGCMGCCGCGGTAA-3’) [24] to 806RB (5’-GGACTACNVGGGTWTCTAAT-3’) [32, 33] on an Illumina MiSeq platform (Illumina Inc., San Diego, CA) as previously described [9]. Negative controls were included at every step during extraction and sequencing and verified to contain <1% of total ASVs for all bacterial taxa. The absolute abundance of contaminants from the negative control data is provided (S1 Table).

### Data analysis

Sequences were processed and analyzed using a Quantitative Insights Into Microbial Ecology 2 (QIIME 2) v 2021.8 pipeline [34]. The raw sequences were uploaded to NCBI Sequence Read Archive under project number PRJNA922951. Briefly, the sequences were demultiplexed and the ASV table was created using DADA2 [35]. Prior to downstream analysis, sequences assigned as chloroplast, mitochondria, and low abundance ASVs, containing less than 0.01% of the total reads in the dataset were removed. The criteria for removing low abundance ASVs was to remove those that were not present in at least 50% of samples from at least one group. Such ASVs are considered rare taxa, and therefore unlikely to be biologically meaningful.

Prevalence-based filtering of putative contaminant ASVs was performed using the R package decontam (v0.99.1) [36]. A DNA extraction blank contemporaneously generated and processed in parallel with biological samples was used as negative control in the filtering procedure. ASV tables were used as the input for the isContaminant() function (pss, method = “prevalence”, neg = “is.neg”, threshold = 0.5). The table with the contaminants generated was visualized with ggplot2, and contaminants were then filtered from the ASV table for downstream analysis. All samples were then rarefied to even sequencing depth, based on the lowest read depth of samples, to 2,835 sequences per sample, to correct for unevenness between samples. At this sequencing depth, the samples had already reached a plateau in number of observed ASVs, indicating that deeper sequencing would be unlikely to change the results of the analysis.

Alpha diversity was measured with the Chao1 (richness), Shannon diversity, and observed ASVs metrics within QIIME2 to compare species richness and evenness amongst the control, unaffected, and ulcerated eyes. Beta diversity (bacterial community composition) was evaluated with the weighted and unweighted phylogeny-based UniFrac [37] distance metric to measure similarity between samples and visualized using Principal Coordinate Analysis (PCoA) plots, generated within QIIME2 to visualize clustering. Bray Curtis was utilized to consider the abundance of the different taxa without analyzing phylogenetical information.

### Statistical analysis

An Analysis of Similarity test (ANOSIM) within PRIMER 7 software package (PRIMER-E Ltd., Luton, UK) was used to analyze significant differences in microbial communities between groups. Specifically, Aitchison, Bray-Curtis, unweighted, and weighted UniFrac distance matrices were analyzed. Microbial communities compared by ANOSIM have an R statistic near 1 when they are different and near 0 when they are similar in composition.

Differences in the relative abundance of bacterial taxa between eyes amongst the three groups were investigated. Kruskal-Wallis tests followed by Dunn’s multiple comparison post-tests were performed to compare ulcerated and unaffected eyes to healthy controls. Wilcoxon matched-pairs signed-rank tests were performed against ulcerated and unaffected eyes (Prism v.9, Graphpad Software Inc.) and adjusted for multiple comparison using Benjamini and Hochberg’s False Discovery Rate [38] at each taxonomic level and a Q value < 0.05 was considered statistically significant.

## Results

### Participants

Following owner consent, samples were collected from 30 eyes of 15 horses with unilateral UK (15 ulcerated eyes and 15 unaffected fellow eyes), as well as 15 randomly selected eyes from healthy horses serving as a normal control for a total of 45 clinical samples.

Twelve of 15 horses (80%) were being treated within two weeks of presentation to the Texas A&M Ophthalmology Service with topical antibiotics and antifungals from varying pharmaceutical companies and compounding pharmacies, including gentamicin sulfate ophthalmic ointment, ciprofloxacin 0.3% ophthalmic solution, ofloxacin 0.3% ophthalmic solution, neomycin-polymixin-bacitracin ophthalmic ointment, terramycin ophthalmic ointment, erythromycin 0.5% ophthalmic ointment, tobramycin 0.3% ophthalmic ointment, miconazole 1% ophthalmic ointment, voriconazole 1% ophthalmic solution. For the 15 eyes with UK, corneal cytology was obtained along with culture-based techniques, including aerobic bacterial and fungal culture to identify infectious microorganisms and aid in a definitive diagnosis (Table 2). Fifteen bacterial isolates in total were cultured amongst the 15 eyes with UK (Table 2). The most commonly cultured bacteria were *Staphylococcus* spp. in 4/15 eyes (26.7%) and *Streptococcus* spp. in 4/15 eyes (26.7%). There were eight fungal isolates cultured, with *Aspergillus* spp. as the most cultured in 3/8 eyes (37.5%). Four out of fifteen horses had no growth on bacterial or fungal cultures, and two of these four horses also did not have microorganisms identified on cytology despite clinical signs consistent with corneal infection (Table 2).

**Table 2:** Study population: Corneal cytology, aerobic bacterial culture, and fungal culture results sampled from the ulcerated eye of horses with a clinical diagnosis of UK.

Study Population Horse	Corneal Cytology	Corneal Aerobic Bacterial Culture	Fungal Culture
**1**	Extracellular bacteria	*Micrococcus* spp., *Staphylococcus* spp., *Streptococcus* spp.	*Aspergillus* spp., *Penicillium* spp., *Streptomyces* spp.
**2**	Intracellular bacteria & fungal hyphae	No growth	No growth
**3**	Extracellular bacteria	Gram + rod, Gram + cocci	No growth
**4**	No organisms identified	No growth	No growth
**5**	Intracellular bacteria	*Streptococcus* spp.	No growth
**6**	Extracellular bacterial	No growth	No growth
**7**	Intracellular bacterial	*Pantoea* spp., *Staphylococcus* spp.	*Alternaria* spp.
**8**	No organisms identified	*Staphylococcus* spp.	*Cladosporium* spp.
**9**	Fungal hyphae	No growth	*Papulaspora* spp.
**10**	Fungal hyphae	No growth	*Aspergillus* spp.
**11**	Fungal hyphae	No growth	*Aspergillus* spp.
**12**	No organisms identified	No growth	No growth
**13**	Intracellular bacterial & fungal hyphae	*Moraxella* spp., *Rothia* spp., *Streptococcus* spp.	*Papulaspora* spp.
**14**	No organisms identified	*Streptococcus* spp.	*Paecilomyces* spp.
**15**	Fungal hyphae & microconidia	*Bacillus* spp., *Staphylococcus* spp., *Stenotrophomonas* spp.	*Fusarium* spp.

### Sequence analysis

Eight amplicon sequence variants were identified as environmental contaminants in the negative sample control (unused swab and 0.2 ml 0.5% tetracaine), which were excluded during data analysis.

All 45 clinical samples collected (conjunctival swabs from 45 eyes of 30 horses) were positive for PCR amplification and yielded sufficient quality sequences. A total of 194,533 sequences were amplified (Min 1,871; Max 4,320; Median 4,320; Mean 4,228.97; SD 412.28) and rarefied to a sequencing depth of 2,835 sequences per sample following quality filtering for data analysis (S1 Fig). Data were used to define the relative abundance of bacteria for each individual sample.

### Species richness and diversity

Samples from control eyes, eyes with UK, and fellow unaffected eyes were compared. The three alpha diversity metrics used included observed amplicon sequence variants (ASVs), which provides insight into the richness of the microbial communities present, Shannon, which considers both abundance and evenness, and Chao1, which estimates richness (diversity) at full sequencing coverage. (Table 3).

**Table 3:** Summary of alpha diversity indices at a depth of 2,835 sequences per sample for control, ulcerated and unaffected eyes.

	Control	Ulcerated	Unaffected	Control vs. Ulcerated		Control vs. Unaffected		Ulcerated vs. Unaffected		Control vs. Ulcerated vs. Unaffected	
	Median (Range)	Median (Range)	Median (Range)	P*	Q***	P*	Q***	P**	Q***	P*	Q***
**Observed ASVs**	345 (102–619)	201 (33–419)	397 (72–622)	0.373	0.560	0.627	0.627	**0.030**	**0.045**	0.065	0.098
**Shannon**	7.1 (3.5–8.8)	6.6 (1.6–8.1)	7.8 (3.9–8.9)	0.906	0.906	0.422	0.627	0.135	0.135	0.129	0.129
**Chao1**	366.3 (26.8–818.3)	219 (34.5–599)	488.6 (3.4–769.7)	0.346	0.560	0.464	0.627	**0.018**	**0.045**	**0.038**	0.098

*: P-values based on Kruskal-Wallis test

**: P-values based on Wilcoxon signed-rank test

***: Q-values adjusted based on the Benjamini & Hochberg False discovery rate

**Fig 1:**
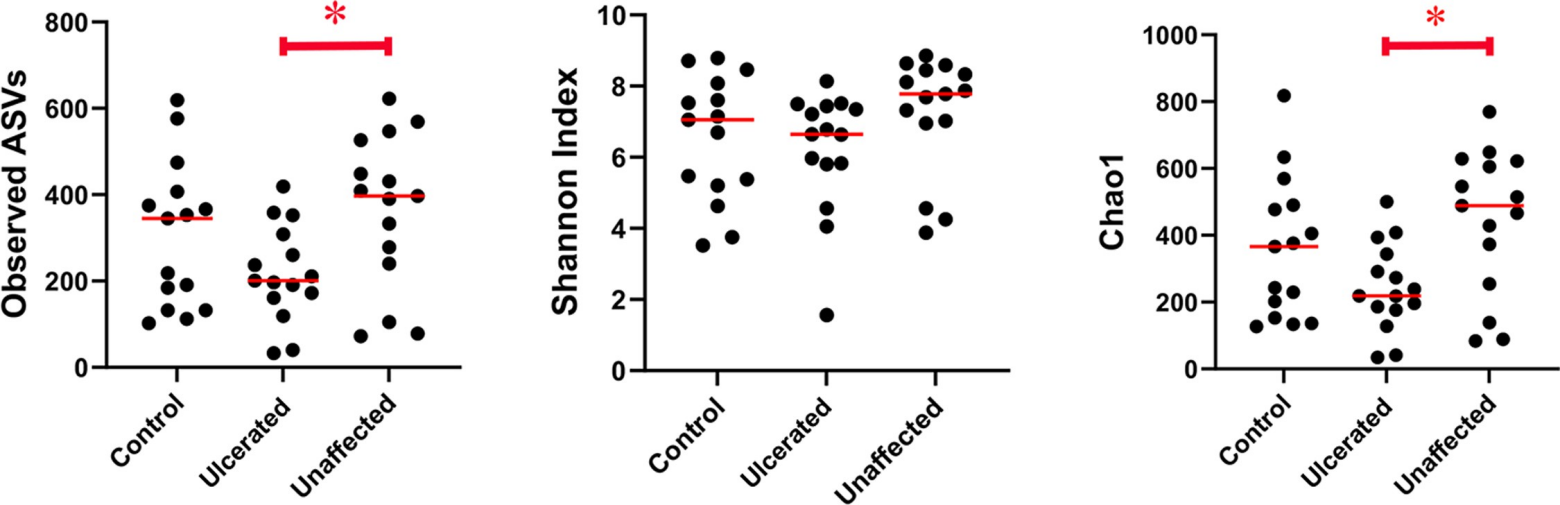
Alpha diversity scatter plots and statistical evaluation of 16S rRNA sequences from ulcerated eyes and unaffected fellow eyes of horses with UK, and healthy control horses. Each dot corresponds to one of 45 eyes from 30 horses. The ocular surface of ulcerated eyes had significantly decreased species richness compared with unaffected fellow eyes (Observed ASVs q = 0.045). The ocular surface of ulcerated eyes had significantly decreased species richness at full sequence coverage compared with unaffected fellow eyes (Chao1 q = 0.045) with no differences in evenness or abundance of species (Shannon q = 0.135).

The ocular surface of ulcerated eyes had significantly decreased species richness and diversity compared with unaffected fellow eyes (Observed ASVs, Chao1), with no differences in abundance or evenness of species (Shannon) (Table 3 and Fig 1).

### Microbial community structure

**Fig 2:**
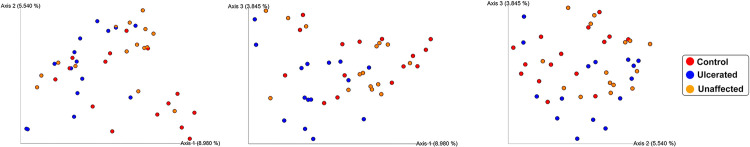
Principal coordinate analysis (PCoA) 2D plots of unweighted UniFrac distance matrices between control (red), ulcerated (blue) and unaffected (orange) eyes of horses. Each dot represents the microbial composition of one eye. Clustering was observed indicating beta diversity was significantly different between either eye of horses with unilateral UK and healthy controls (control vs. unaffected, p = 0.035; control vs. ulcerated, p = 0.003) as well as between eyes of horses with unilateral UK (unaffected vs. ulcerated, p = 0.016).

Beta diversity measures (Bray Curtis, weighted UniFrac) indicated there was a significant difference in community structure and relative abundance of taxa detected between ulcerated eyes of horses with UK and healthy control eyes (Bray Curtis: R = 0.205, p = 0.002; weighted UniFrac: R = 0.183, p = 0.001) (S2 Fig). A significant difference in microbial communities was observed between all three groups with unweighted UniFrac Analysis of Similarities (ANOSIM) (R = 0.225, p = 0.003 for control vs. ulcerated eyes; R = 0.098, p = 0.035 for control vs. unaffected fellow eyes; R = 0.113, p = 0.016 for ulcerated vs. unaffected fellow eyes) and evidenced by clustering in the Principal Coordinate Analysis plot (PCoA) (Fig 2).

### Microbial community composition

**Fig 3:**
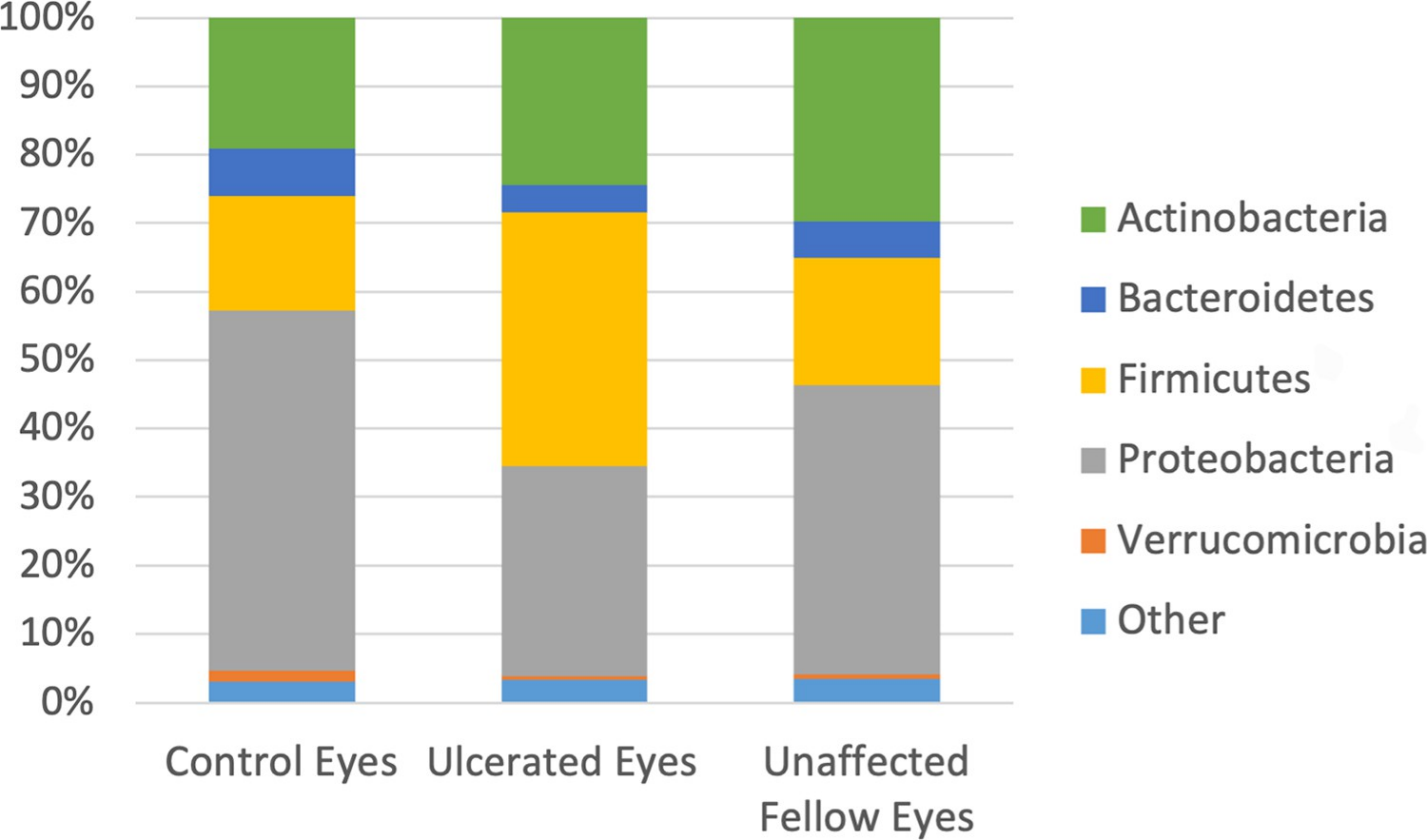
Composition of the equine ocular surface in control, ulcerated and unaffected eyes. Relative abundance of a taxa annotated to the level of bacterial phylum. The bars represent the mean percentage totaling 100% for each group. Taxa < 1% mean relative abundance are grouped into “other”.

**Fig 4:**
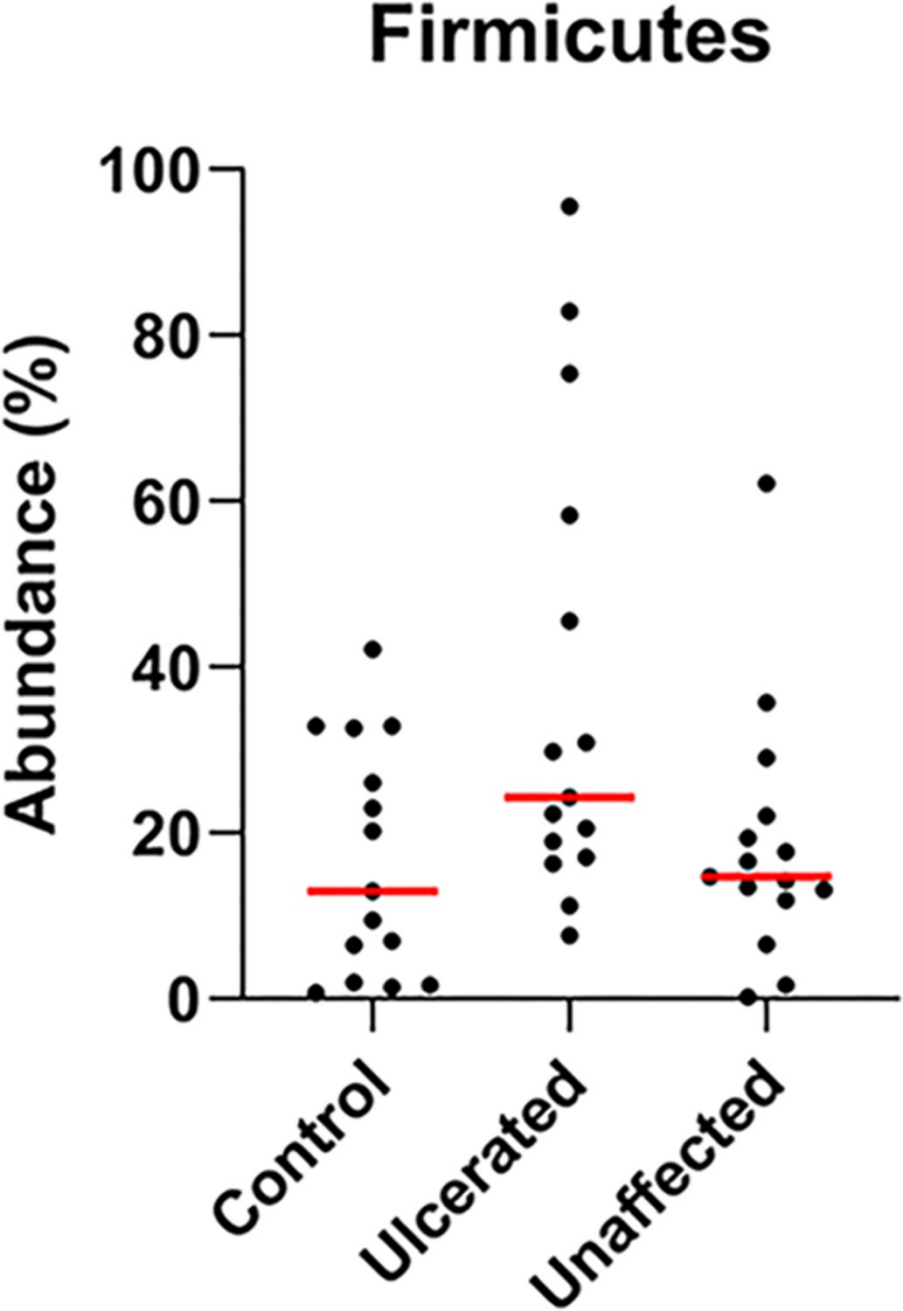
Scatter plot of the relative abundance of bacterial phyla Firmicutes. Although no significant differences were detected between control, ulcerated, and unaffected eyes (q = 0.216), there is a trend for increased relative abundance of Firmicutes in ulcerated eyes (p = 0.043).

There were significant differences in bacterial taxa abundance between ulcerated eyes, unaffected fellow eyes, and control eyes. Data from all 45 eyes were averaged to describe the bacterial taxa composition of each group. A total of 15 bacterial phyla were detected in all three groups with 10 of the taxa representing <1% mean relative abundance. The remaining five phyla (Firmicutes, Proteobacteria, Actinobacteria, Bacteroidetes, Verrucomicrobia) represented the majority in all three groups. The most common phyla were Proteobacteria (control 52.57%, ulcerated 30.66%, unaffected 42.18%), Firmicutes (control 16.75%, ulcerated 37.10%, unaffected 18.55%), Actinobacteria (control 19.1%, ulcerated 24.42%, unaffected 29.5%), and Bacteroidetes (control 6.94%, ulcerated 4%, unaffected 5.43%) (S2 Table). Firmicutes were more abundant in ulcerated eyes (p = 0.026); however, when p-values were corrected for false discovery rate no significant changes were detected at the phylum level (Table 4 and Figs 3 and 4).

**Table 4:** Taxa present from the ocular surface of control, ulcerated, and unaffected eyes of horses with significant (q<0.05) or trending (p<0.05) alterations. Median relative percentages and ranges of the most abundant bacterial groups, annotated to the level of phylum, class, family, and genus based on sequencing of the 16S rRNA.

Taxon Phylum ‐Class ‐‐Family *‐‐‐Genus*	Control Eyes	Ulcerated Eyes	Unaffected Fellow Eyes	Control vs. Ulcerated		Control vs. Unaffected		Ulcerated vs. Unaffected		Control vs. Ulcerated vs. Unaffected	
	Median (Range)	Median (Range)	Median (Range)	P *	Q ***	P *	Q***	P **	Q***	P*	Q***
**Firmicutes**	13 (0.7–42.2)	24.3 (7.6–95.6)	14.7 (0.3–62.1)	0.078	0.484	1.00	1.00	**0.026**	0.384	**0.043**	0.216
‐‐Clostridiaceae	0.35 (0–1.59)	0 (0–0.95)	0.49 (0–1.09)	0.126	0.421	1.00	1.00	**0.043**	0.728	**0.022**	0.156
‐‐‐Unclassified Clostridiales	0.95 (0–10.55)	0 (0–0.92)	0.32 (0–3.28)	**0.014**	0.165	0.276	0.875	0.307	0.841	**0.017**	0.142
‐‐‐*Unclassified Clostridiales*	0.95 (0–10.55)	0 (0–0.92)	0.32 (0–3.28)	**0.014**	0.141	0.276	0.718	0.307	0.794	**0.017**	0.082
‐‐Ruminococcaceae	1.09 (0–13.58)	0 (0–2.93)	0.39 (0–5.4)	**0.006**	0.096	0.812	1.00	0.147	0.804	**0.007**	0.083
‐‐‐*Unclassified Ruminococcaceae*	0.95 (0–11.71)	0 (0–2.5)	0.25 (0–4.37)	**0.008**	0.127	0.690	1.00	0.224	0.794	**0.011**	0.077
‐Bacilli	3 (0.49–22.61)	22.05 (7.05–95.56)	12.03 (0.25–54.36)	**0.001**	**0.004**	0.212	0.624	**0.022**	0.667	**0.001**	**0.007**
‐‐Staphylococcaceae	0.35 (0–2.86)	3.63 (0–41.98)	2.79 (0.07–18.59)	**0.001**	0.096	**0.004**	0.145	0.151	0.804	**0.001**	**0.030**
‐‐‐*Salinicoccus*	0 (0–2.12)	0.6 (0–4.83)	1.06 (0–2.89)	**0.006**	0.119	**0.001**	0.081	0.950	1.00	**0.001**	**0.031**
‐‐‐*Staphylococcus*	0.32 (0–2.12)	1.73 (0–38.8)	1.55 (0–14.6)	**0.009**	0.127	**0.044**	0.305	0.090	0.656	**0.006**	0.061
‐‐‐Streptococcaceae	0.14 (0–2.12)	1.27 (0–95.56)	0.18 (0–2.93)	0.094	0.406	1.00	1.00	**0.012**	0.728	**0.042**	0.160
‐‐‐*Streptococcus*	0.04 (0–2.12)	1.27 (0–95.56)	0.18 (0–2.93)	0.073	0.298	1.00	1.00	**0.012**	0.418	**0.038**	0.114
‐‐Planococcaceae	0.21 (0–1.23)	0.71 (0–3.7)	0.88 (0–6.07)	0.766	1.00	**0.025**	0.380	0.073	0.728	**0.031**	0.160
‐‐Bacillaceae	0.74 (0–14.57)	1.8 (0–36.68)	2.19 (0-04-39.22)	0.214	0.562	**0.035**	0.380	0.978	1.00	**0.034**	0.160
‐‐Aerococcaceae	0 (0–1.06)	0.92 (0–4.16)	0.85 (0–2.54)	**0.027**	0.246	0.070	0.483	0.514	0.866	**0.018**	0.142
‐‐‐*Facklamia*	0 (0–0.92)	0.32 (0–1.94)	0.28 (0–1.02)	**0.025**	0.160	0.056	0.367	0.367	0.801	**0.015**	0.082
**Proteobacteria**	50.1 (22.3–96.4)	28.5 (3–90)	38.7 (10.8–94.5)								
‐‐Xanthomonadaceae	0.35 (0–2.68)	1.2 (0.32–6.31)	1.09 (0–3.46)	**0.021**	0.227	0.094	0.541	0.454	0.866	**0.017**	0.142
‐‐‐*Unclassified Xanthomonadaceae*	0 (0–1.02)	0.42 (0–2.4)	0.28 (0–1.31)	0.196	0.503	0.926	1.00	**0.036**	0.418	0.182	0.284
‐‐Sphingomonadaceae	3.39 (0.32–9.66)	0.74 (0–3.77)	2.01 (0.11–7.02)	**0.040**	0.266	0.603	1.00	0.083	0.728	**0.047**	0.160
‐‐‐*Sphingomonas*	3.07 (0.18–8.15)	0.39 (0–1.62)	1.23 (0–4.13)	**0.003**	0.119	0.490	1.00	**0.038**	0.418	**0.004**	0.060
‐‐‐Methylobacteriaceae	0.53 (0–1.87)	0 (0–1.59)	0.11 (0–1.27)	**0.045**	0.273	0.440	0.978	0.398	0.865	**0.050**	0.160
‐‐‐*Methylobacterium*	0.53 (0–1.38)	0 (0–1.59)	0.04 (0–0.63)	0.057	0.261	0.110	0.481	0.610	0.841	**0.037**	0.111
‐‐‐*Unclassified Oxalobacteraceae*	0.21 (0–1.13)	0 (0–0.71)	0.32 (0–1.13)	0.073	0.298	0.740	1.00	**0.006**	0.418	**0.002**	0.060
‐‐‐*Unclassified Moraxellaceae*	0.35 (0–7.8)	0.11 (0–2.22)	0.39 (0–3.56)	1.00	1.00	1.00	1.00	**0.045**	0.457	0.282	0.381
**Bacteroidetes**	5 (0.5–18.1)	3 (0–8.8)	4.9 (0.1–14.7)								
‐Bacteroidia	2.08 (0–14.71)	0.42 (0–4.06)	0.53 (0–6.03)	**0.033**	0.256	0.177	0.624	0.489	0.837	**0.031**	0.136
‐‐Unclassified Bacteroidales	0.71 (0–9.84)	0 (0–2.22)	0.35 (0–3.63)	**0.003**	0.096	0.132	0.538	0.401	0.865	**0.004**	0.083
‐‐‐*Unclassified Bacteroidales*	0.71 (0–9.84)	0 (0–2.22)	0.35 (0–3.63)	**0.002**	0.119	0.132	0.510	0.401	0.418	**0.004**	0.060
‐‐‐*Unclassified RF16*	0.35 (0–1.62)	0 (0–0.18)	0 (0–0.46)	**0.006**	0.119	0.218	0.664	0.142	0.745	**0.008**	0.067
‐‐‐*Hymenobacter*	0.25 (0–1.16)	0 (0–0.25)	0.07 (0–1.02)	**0.006**	0.119	1.00	1.00	**0.037**	0.418	**0.005**	0.060
**Actinobacteria**	11.9 (1.3–70.7)	25.6 (1–49.8)	29.2 (2.6–81.1)								
‐‐Dermabacteraceae	0 (0–0.56)	0.71 (0–3)	0.88 (0–4.13)	**0.033**	0.246	**0.001**	**0.048**	0.229	0.804	**0.001**	**0.030**
‐‐‐*Brachybacterium*	0 (0–0.56)	0.71 (0–3)	0.88 (0–4.13)	**0.029**	0.166	**0.001**	0.079	0.229	0.794	**0.001**	**0.031**
‐‐‐Intrasporangiaceae	0.04 (0–1.27)	1.62 (0–8.43)	1.31 (0.04–8.96)	**0.005**	0.096	**0.004**	0.145	0.847	0.976	**0.001**	**0.041**
‐‐‐*Unclassified Intrasporangiaceae*	0.04 (0–0.56)	1.59 (0–7.2)	0.6 (0.04–7.69)	**0.002**	0.119	**0.003**	0.090	0.804	0.942	**0.001**	**0.031**
‐‐‐Unclassified Actinomycetales	0.42 (0–2.82)	0.28 (0–1.87)	0.95 (0–3.35)	1.00	1.00	0.442	0.978	**0.026**	0.728	0.091	0.226
‐‐‐*Unclassified Actinomycetales*	0.42 (0–2.82)	0.28 (0–1.87)	0.95 (0–3.35)	1.00	1.00	0.442	0.949	**0.026**	0.418	0.091	0.192
‐‐‐*Unclassified Micrococcaceae*	0 (0–0.25)	0 (0–1.55)	0.32 (0–1.09)	0.281	0.539	**0.002**	0.081	0.350	0.801	**0.003**	0.060
‐‐‐*Unclassified Nocardioidaceae*	0.14 (0–0.78)	0.71 (0–1.76)	0.42 (0.2.01)	**0.026**	0.160	0.077	0.435	0.550	0.841	**0.018**	0.082
‐‐‐*Unclassified Pseudonocardiaceae*	0.53 (0–1.76)	0 (0–1.66)	0.14 (0–1.34)	**0.022**	0.160	0.654	1.00	0.230	0.794	**0.028**	0.096

*: P-values based on Kruskal-Wallis test

**: P-values based on Wilcoxon signed-rank test

***: Q-values adjusted based on the Benjamini & Hochberg False discovery rate

**Fig 5:**
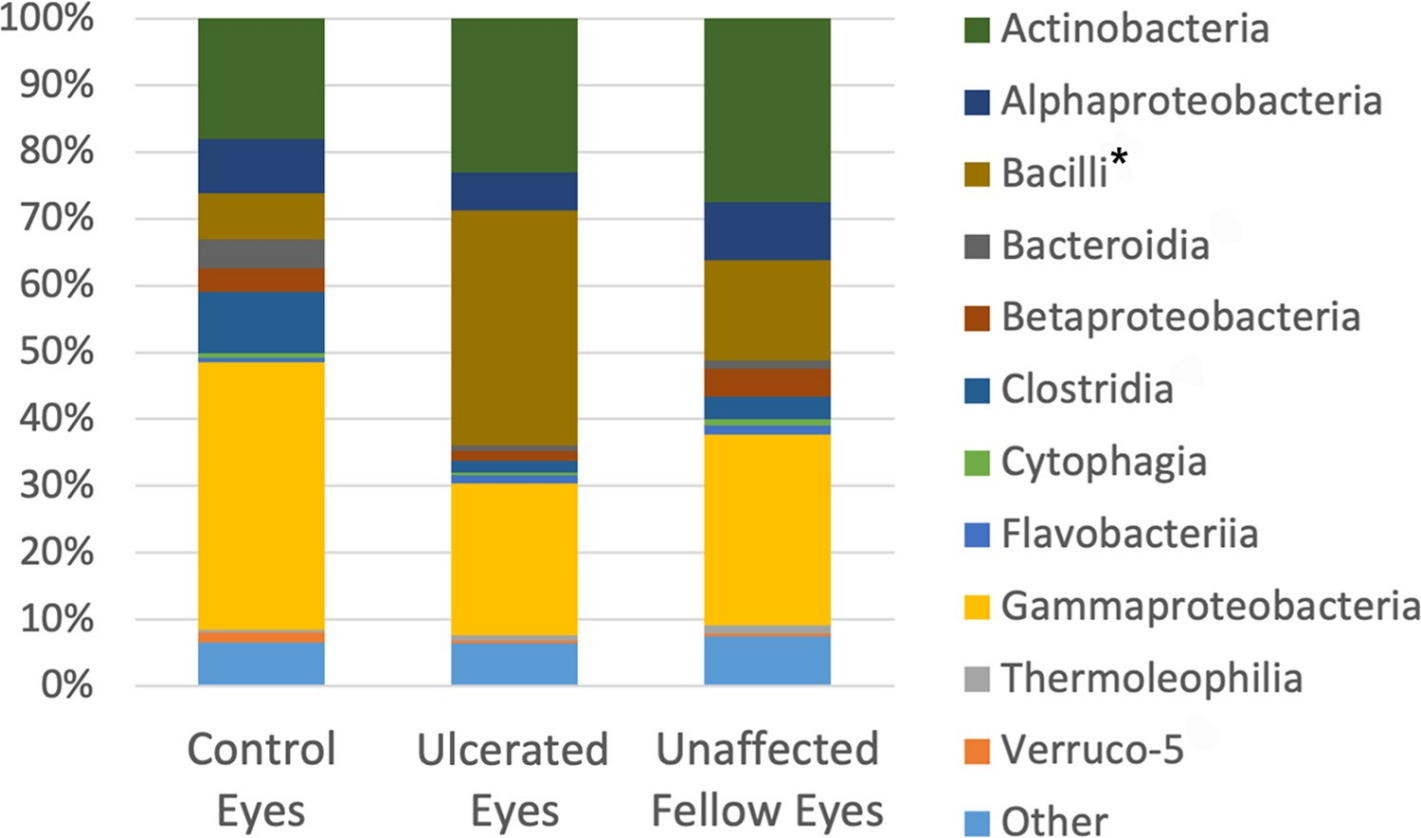
Composition of the equine ocular surface in control, ulcerated and unaffected eyes. Relative abundance of a taxa annotated to the level of bacterial class. The bars represent the mean percentage totaling 100% for each group. Taxa < 1% mean relative abundance are grouped into “other”. Note significant differences in abundance between groups (q < 0.05) annotated by (*).

A total of 31 classes were detected and 11 were present in all three groups (Verruco-5, Thermoleophilia, Gammaproteobacteria, Flavobacteriia, Cytophagia, Clostridia, Betaproteobacteria, Bacteroidia, Bacilli, Alphaproteobacteria, Actinobacteria), with 20 of the taxa representing <1% mean relative abundance. The most identified classes were Gammaproteobacteria (control 40.17%, ulcerated 22.85%, unaffected 28.58%), Actinobacteria (control 18.11%, ulcerated 23.1%, unaffected 27.53%), and Bacilli (control 6.86%, ulcerated 35.2%, unaffected 15.09%) (S2 Table). Bacilli were significantly more abundant in ulcerated eyes compared to healthy control eyes (q = 0.004) (Table 4 and Fig 5).

**Fig 6:**
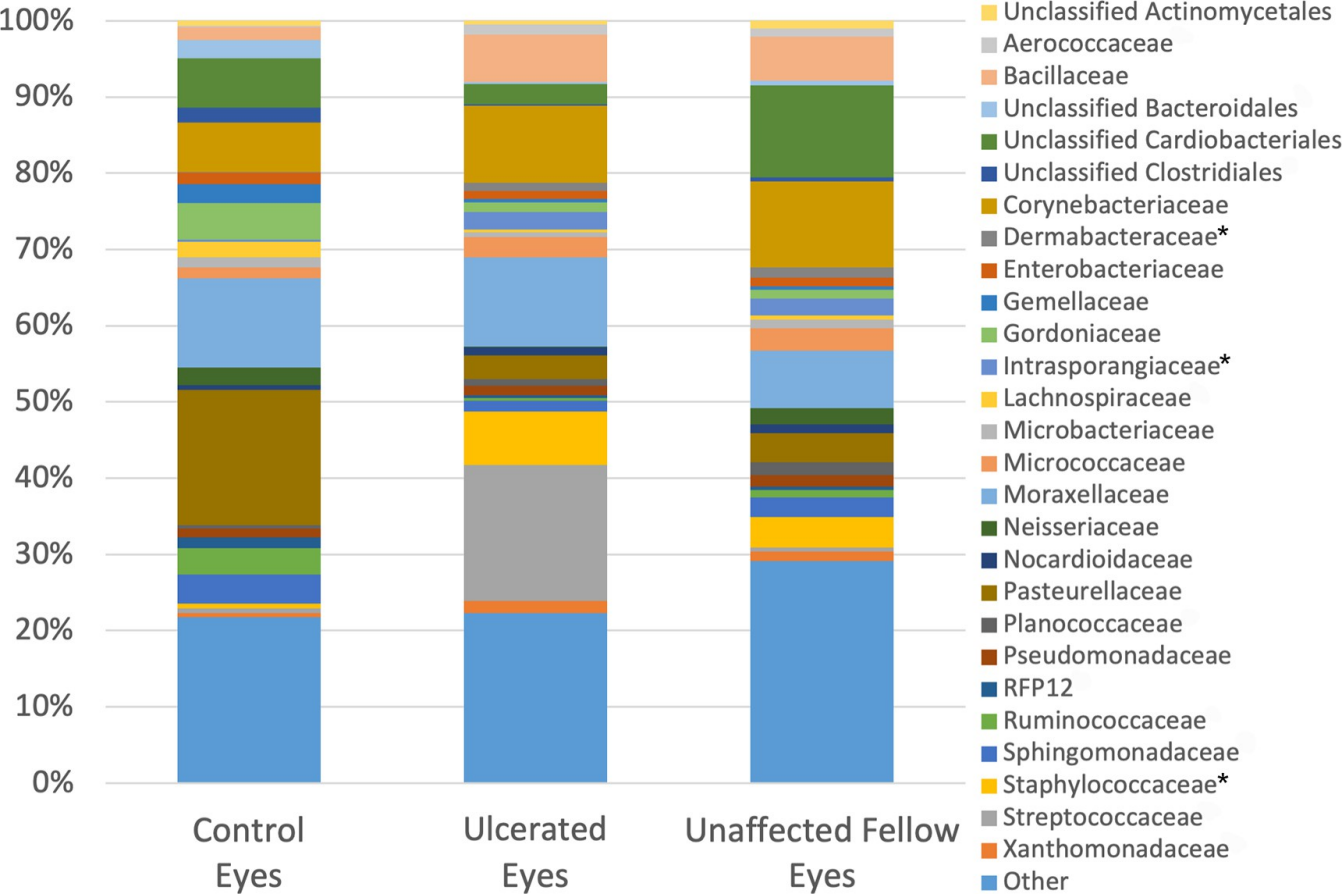
Composition of the equine ocular surface in control, ulcerated and unaffected eyes. Relative abundance of a taxa annotated to the level of bacterial family. The bars represent the mean percentage totaling 100% for each group. Taxa < 1% mean relative abundance are grouped into “other”. Note the significant differences in abundance between groups (q < 0.05) annotated by (*).

A total of 97 families were detected and 27 were present in all three groups with 60 of the taxa representing <1% mean relative abundance. The most identified families were Moraxellaceae (control 11.64%, ulcerated 11.73%, unaffected 7.52%), Corynebacteriaceae (control 6.47%, ulcerated 10.1%, unaffected 11.28%), an unclassified order of Cardiobacteriales (control 6.56%, ulcerated 2.67%, unaffected 12.08%), Pasteurellaceae (control 17.73%, ulcerated 3.1%, unaffected 3.76%) (S2 Table). Notably within the phylum Firmicutes and class Bacilli, Staphylococcaceae were more abundant in ulcerated and unaffected eyes compared to healthy control eyes (q = 0.030) (Table 4 and Fig 6). Within the phylum Actinobacteria, Dermabacteraceae and Intrasporangiaceae were more abundant in ulcerated and unaffected eyes compared to controls (q = 0.030 and 0.041, respectively).

A total of 130 genera were detected and 24 were present in all three groups with 106 of the taxa representing <1% mean relative abundance. The most identified genera were *Acinetobacter* (control 1.8%, ulcerated 10.5%, unaffected 5.57%), an unclassified genera of Pasteurellaceae (control 17.41%, ulcerated 3.01%, unaffected 3.52%), *Corynebacterium* (control 6.47%, ulcerated 10.1%, unaffected 11.28%), *Gordonia* (control 4.78%, ulcerated 1.27%, unaffected 1.15%), and an unclassified genera of Cardiobacteriales (control 6.56%, ulcerated 2.67%, unaffected 12.08%). *Salinicoccus* spp. from the family Staphylococcaceae were significantly increased in ulcerated and unaffected eyes compared to controls (q = 0.031). Among Acinobacteria, *Brachybacterium* spp. and an unclassified genus of Intrasporangiaceae were also elevated among ulcerated and unaffected eyes compared to controls (q = 0.031 and 0.031, respectively) (Table 4).

**Fig 7:**
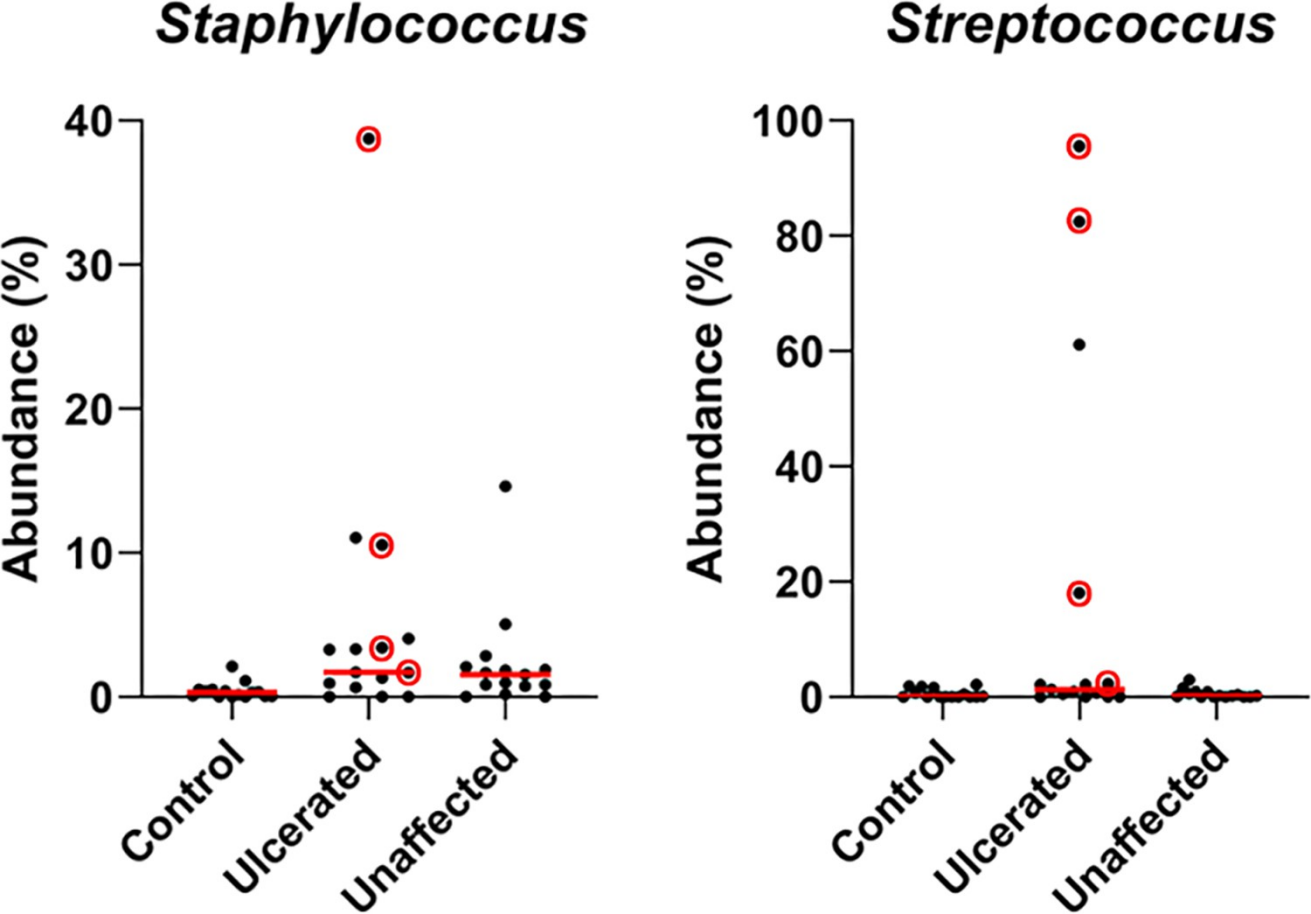
Scatter plots of the relative abundance of bacterial genera *Staphylococcus* and *Streptococcus*. Although no significant differences were detected between control, ulcerated, and unaffected eyes for *Staphylococcus* and *Streptococcus* spp. (q = 0.061 and 0.114, respectively), there is a trend for increased relative abundance of *Staphylococcus* spp. in ulcerated and unaffected eyes compared to controls (p = 0.009 and 0.044, respectively), and increased relative abundance of *Streptococcus* spp. in ulcerated eyes (p = 0.042). Note the ulcerated eyes that isolated *Staphylococcus* or *Streptococcus* spp. on aerobic culture are denoted by a red circle.

*Staphylococcus* and *Streptococcus* spp. were the two most common bacteria cultured from ulcerated eyes in this study, consistent with previous reports of equine infectious ulcerative keratitis (Table 2). Fig 7 shows a trend in increased relative abundance of *Staphylococcus* and *Streptococcus* spp. among ulcerated eyes (p = 0.006 and 0.038, respectively); however, when p-values were corrected for false discovery rate no significant changes were detected (Table 4 and Fig 7). Two of four ulcerated eyes culture-positive for *Staphylococcus* spp. and three of four ulcerated eyes culture-positive for *Streptococcus* spp. had increased relative abundance of those genera on 16S sequencing (Fig 7).

## Discussion

Previous microbiome studies have demonstrated that the equine ocular surface contains a diverse bacterial microbiota and mycobiota [5, 6], which is exemplified in the present study. The most common phyla colonizing the eyes in all three groups in this study, Proteobacteria, Firmicutes, Actinobacteria, and Bacteroidetes, aligns with a previous equine ocular surface microbiome study using NGS, and is similar to the most commonly noted microorganisms found using molecular-based techniques in human, canine and feline ocular microbiome studies [3–5, 39–41]. The most relatively abundant bacterial families identified in the present study are also comparable to previous equine ocular microbiome research, with minor variations: Moraxellaceae, an unclassified order of Cardiobacteriales, Pasteurellaceae, and Corynebacteriaceae [5].

The purpose of the present study was to compare the equine ocular surface microbiota of eyes free of ocular disease to eyes with ulcerative keratitis and the fellow unaffected eye. In doing so, we identified significant decreases in species richness and diversity of ulcerated eyes compared to unaffected fellow eyes, with no difference in the abundance or evenness of species. Beta diversity measures revealed a significant difference in community structure between all three groups. This would suggest dysbiosis and bacterial overgrowth of pathogenic species. A human study examining alterations in the ocular microbiome in patients with dry eye showed significant differences in beta diversity between control eyes and eyes with autoimmune dry eye syndrome [42]. Many studies have shown a reduction in the intestinal microbiome diversity in patients with Crohn’s disease, type 1 diabetes mellitus, allergies, multiple sclerosis, among other autoimmune diseases. Furthermore, a study examining the ocular microbiome using NGS showed marked changes in beta diversity between control eyes and eyes with traumatic corneal ulcers in human patients, with a more enriched flora in control patients than those with traumatic ulcers, suggesting overgrowth of pathogenic bacteria [43].

The ocular surface is an exposed mucosa and a component of the mucosal immune system. As well, it is exposed to external stressors, including microbial pathogens, environmental irritants, desiccation, among others, but in healthy eyes, maintenance of homeostasis precludes disease [44]. Alterations of the ocular microbiota may lead to immune compromise, termed dysbiosis, which lead to disruption of ocular surface homeostasis and subsequent disease [44]. It is possible that intrinsic factors, such as sex, age or genetic deficiencies, as well as extrinsic factors, such as environment (climate, season, weather, housing) may play a role in the pathogenesis of disease [14, 45, 46], although a recent study investigating the microbiome and mycobiome in healthy horses did not identify age, breed or sex as risk factors [17]. Gut dysbiosis has been associated with immune-mediated diseases in humans [7, 42]. Some authors have proposed that specific gut bacteria may contribute to modulation of T-regulatory cells, resulting in an increased susceptibility to immune-mediated processes [7]. The investigation of gut microbiota composition in human patients with acute anterior uveitis (AAU) had a unique fecal metabolic phenotype compared with controls [47]. Phenotypic discrepancies may play a role in ocular microbial differences between one horse to another.

There were no notable changes in the relative abundance of bacterial phyla or families over time in healthy equine eyes in a previous ocular microbiome study [5], which exemplifies the ability of the ocular surface microbiome to maintain stability when not faced with disease. In the present study, observable differences were seen in the relative abundance of various bacterial phyla, classes, families, and genera between control eyes, eyes with ulcerative keratitis and unaffected fellow eyes. As stated, there were statistically significant increases in the mean relative abundance of the class Bacilli in ulcerated eyes compared to healthy control eyes. This change speaks to the effects of dysbiosis and propensity of a commensal microorganism to lead to deleterious complications. Interestingly, a previous study identified that a co-housed group of healthy horses with no ocular disease had a higher frequency of gram-positive Bacilli compared to other groups, with the proposed explanation being due to the presence of Bacilli within that particular environment [17]. These species contain a wide array of genes encoding extracellular factors such as degradative enzymes (phospholipases, proteases, and chitinases), cytotoxic proteins (hemolysins, enterotoxins, and cytotoxins) and cell surface proteins that contribute to corneal compromise [48]. The population of horses in this study varied in their home environments, with 15 client-owned horses with ulcerative keratitis originating from different locations across Texas, while the healthy control horses were housed together on either pasture or in stables on the University’s campus.

The families Staphylococcaceae, Dermabacteraceae, and Intrasporangiaceae were significantly elevated in both ulcerated and unaffected eyes compared to controls, suggesting bacterial overgrowth. Their surgencies in both ulcerated and unaffected fellow eyes suggest changes in the ocular microbiome of these horses due to unidentified ocular pathologies or other factors may contribute to disease. Staphylococcaceae and Streptococcaceae families are commonly cultured from the healthy and diseased equine ocular surface and were the most frequently cultured bacteria in the present study [12–26]. The lack of detection of Dermabacteraceae or Intrasporangiaceae taxa from our culture results and overall low relative abundance of Staphylococcaceae and Streptococcaceae on 16S sequencing shows the vast array of microorganisms not being detected with culture-based techniques alone. Specifically, four out of 15 horses in this study had no growth on aerobic bacterial and fungal cultures, but the use of NGS detected organisms in all samples.

Focusing at the genus level, NGS detected an elevation in relative abundance of *Staphylococcus* and *Streptococcus* spp. among select ulcerated and unaffected eyes; however, this did not reach significance. As well, *Pseudomonas aeruginosa* is a species commonly linked to bacterial keratitis in veterinary species but in this study, it was not cultured in our study population and had a low relative abundance on NGS. Comparing our NGS and culture results among ulcerated eyes shows some discrepancy between the two tests (Fig 7). These findings support potential future applicability of NGS’s highly sensitive microbiological detection modality for ocular infections in veterinary species, which may help tailor specific antimicrobial therapy for vision-threatening diseases.

There are limitations to this study, including a small and diverse equine population, especially those representing a varied sampling of privately-owned horses with different housing environments for the horses with ulcerative keratitis. Our goal is to obtain uniformity when it comes to husbandry and patient signalment, but our findings represent what is found in the general equine population compared to a research herd. As mentioned, there is variability in the conclusions drawn about the impact of group variations on the composition of the ocular surface microbiome in horses. Examples that have at times been equivocal include age, sex, season, geography, and environment [14, 17, 45, 46]. In this study, control horses within the research herd were housed in the same environment compared to the heterogenous housing environments for the horses with ulcerative keratitis. The former likely poses a degree of bias and is acknowledged as a limitation if the environment were to impact the ocular surface microbial composition, as was seen in the Hampson *et al*. study, showing that horses housed in the same environment had a higher frequency of gram-positive bacilli compared to other groups [17].

Another possible limitation is the use of conjunctival fornix sampling rather than the corneal ulcer for NGS. A molecular-based human study examining corneal and conjunctival microbiota showed significant differences between the microbial genus and species level [49]. Additionally, the healthy porcine corneal surface had a significantly lower abundance of taxa with compositional differences compared to the conjunctival surface of the same subjects [50]. However, the reproducibility of sampling sites has not been investigated with cases of presumed infectious ulcerated keratitis where microbial overgrowth is likely occurring, and aerobic bacterial culture of the conjunctival fornix was found to be a suitable alternative to direct ulcer sampling in dogs with presumed bacterial keratitis [51]. Sampling the ocular surface with varying pressures and duration can also result in different microflora if only capturing the superficial layer. “Deep” swabbing, using moderate pressure with a dry swab, is recommended over “soft” swabbing, using minimal pressure with a wet swab, for the most representative sample in low biomass tissues for NGS [52]. Since the clinical presentation of ulcerative keratitis in horses is severe, often with marked stromal loss compromising the integrity of the globe, the authors elected to directly sample the ulcer for the gold standard diagnostic test of aerobic culture while performing a “deeper” swab of the conjunctival fornix for NGS to avoid taking multiple samples at a fragile tissue site. Therefore, we presumed our conjunctival samples would be an approximate representation of the infected ocular surface microbiome and acknowledge this limitation of the study. Future studies should investigate the exact location of ocular surface sampling in diseased eyes to better understand its reliability.

An additional limitation inherent to microbiome studies includes the interpretation of relative abundance, which does not consider the absolute bacterial quantities present in a sample, and cannot be obtained through NGS [48]. Quantitative PCR of specific bacteria or fungi can be performed to determine absolute quantities in a sample [5]. NGS detects the presence of organism DNA but cannot determine if it is living. Another limitation of this study is that 12/15 horses presented with topical medications on board for a period of less than 2 weeks. Previous microbiome studies of healthy eyes in various veterinary species have shown that there is no significant change to the ocular microbiome with the use of topical antibiotics [4, 5, 9]. However, the effects of antimicrobials on the ocular microbiome have yet to be elucidated in eyes with ulcerative keratitis and further studies are indicated to draw conclusions.

## Conclusion

This report showed that bacterial community structure was altered in both the ulcerated and unaffected fellow eyes of horses with unilateral ulcerative keratitis compared to control eyes of healthy horses. As well, ulcerated eyes of horses with unilateral UK had decreased species richness and diversity compared to their unaffected fellow eyes. Evidence of dysbiosis was seen in both ulcerated eyes and unaffected fellow eyes. The microbiome may play a role in the cause of UK in horses. Further investigations to determine the role of the microbiome in the pathophysiology of ocular surface disease are warranted.

## Supporting information

S1 Table Absolute abundance of contaminants from the negative control (two unused swabs combined with 0.2 ml 0.5% tetracaine). Number of counts annotated to the level of phylum, class, family, and genus based on sequencing of the 16S rRNA.
(DOCX)

S2 Table Taxa present at >1% mean relative abundance in healthy control eyes, ulcerated eyes, and unaffected fellow eyes. Mean relative percentages and standard deviation of the most abundant bacterial groups, annotated to the level of phylum, class, family, and genus based on sequencing of the 16S rRNA.
(DOCX)

S1 Fig Rarefaction analysis of 16S rRNA gene sequences from horses comparing infected eyes, unaffected fellow eyes, and healthy control eyes. (TIF)

S2 Fig Principal coordinate analysis (PCoA) 2D plots of (A) Aitchison, (B) Bray-Curtis, and (C) weighted UniFrac distance matrices between control (red), ulcerated (blue) and unaffected (orange) eyes of horses. Each dot represents the microbial composition of one eye. Clustering was observed indicating a significant difference in community structure and relative abundance of taxa detected between ulcerated eyes of horses with UK and healthy control eyes (Bray Curtis: R = 0.205, p = 0.002; weighted UniFrac: R = 0.183, p = 0.001). The remaining distance matrices and comparisons showed no difference in community structure (Aitchison: R = 0.03 control vs. unaffected, R = 0.019 control vs. ulcerated, R = 0.039 ulcerated vs. unaffected; Bray-Curtis: R = 0.064 control vs. unaffected, R = -0.011 ulcerated vs. unaffected; weighted UniFrac: R = 0.05 control vs. unaffected, R = 0.018 ulcerated vs. unaffected; p > 0.05).
(TIF)
