# Supplementary material for: Alterations of the bacterial ocular surface microbiome are found in both eyes of horses with unilateral ulcerative keratitis
Source: PLoS One. 2023 Sep 8;18(9):e0291028. doi: 10.1371/journal.pone.0291028 (PMC10490969; doi:10.1371/journal.pone.0291028)
Supplement: S1 Table — Number of counts annotated to the level of phylum, class, family, and genus based on sequencing of the 16S rRNA. (DOCX) [file pone.0291028.s001.docx]

**S1 Table. Absolute abundance of contaminants from the negative control (two unused swabs combined with 0.2 ml 0.5% tetracaine).** Number of counts annotated to the level of phylum, class, family, and genus based on sequencing of the 16S rRNA.

| **Taxon**  **Phylum**  -Class  --Family  ---*Genus* | **Negative Control Sample** |
| --- | --- |
| **Firmicutes** | 7 |
| -Bacilli | 7 |
| --Staphylococcaceae | 7 |
| ---*Staphylococcus* | 7 |
| **Proteobacteria** | 3656 |
| -Alphaproteobacteria | 658 |
| --Phyllobacteriaceae | 658 |
| -Betaproteobacteria | 792 |
| --Alcaligenaceae | 321 |
| ---*Achromobacter* | 321 |
| --Oxalobacteraceae | 471 |
| ---*Unclassified*  *Oxalobacteraceae* | 471 |
| -Gammaproteobacteria | 2206 |
| --Enterobacteriaceae | 2206 |
| **Bacteroidetes** | 636 |
| -Sphingobacteriia | 4 |
| --Sphingobacteriaceae | 4 |
| -Unclassified  Saprospirales | 632 |
| --Chitinophagaceae | 632 |
| ---*Sediminibacterium* | 629 |
| **Actinobacteria** | 21 |
| -Thermoleophilia | 2 |
| --Gaiellaceae | 2 |
| -Actinobacteria | 19 |
| --Propionibacteriaceae | 19 |
| ---*Propionibacterium* | 19 |
