# Supplementary material for: Alterations of the bacterial ocular surface microbiome are found in both eyes of horses with unilateral ulcerative keratitis
Source: PLoS One. 2023 Sep 8;18(9):e0291028. doi: 10.1371/journal.pone.0291028 (PMC10490969; doi:10.1371/journal.pone.0291028)
Supplement: S2 Table — Mean relative percentages and standard deviation of the most abundant bacterial groups, annotated to the level of phylum, class, family, and genus based on sequencing of the 16S rRNA. (DOCX) [file pone.0291028.s002.docx]

**S2 Table. Taxa present at** >**1% mean relative abundance in healthy control eyes, ulcerated eyes, and unaffected fellow eyes.** Mean relative percentages and standard deviation of the most abundant bacterial groups, annotated to the level of phylum, class, family, and genus based on sequencing of the 16S rRNA.

| **Taxon**  **Phylum**  -Class  --Family  ---*Genus* | **Control Eyes** | | **Ulcerated Eyes** | | **Unaffected Fellow Eyes** | |
| --- | --- | --- | --- | --- | --- | --- |
|  | **Mean (%)** | **SD** | **Mean (%)** | **SD** | **Mean (%)** | **SD** |
| **Firmicutes** | 16.75 | 14.05 | 37.10 | 27.98 | 18.55 | 15.17 |
| -Clostridia | 9.21 | 11.56 | 1.77 | 2.09 | 3.33 | 3.53 |
| --Unclassified  Clostridiales | 1.94 | 2.93 | 0.2 | 0.33 | 0.55 | 0.88 |
| *---Unclassified*  *Clostridiales* | 1.94 | 2.93 | 0.2 | 0.33 | 0.55 | 0.88 |
| --Ruminococcaceae | 3.49 | 4.51 | 0.38 | 0.84 | 1.02 | 1.43 |
| ---*Unclassified*  *Ruminococcaceae* | 2.92 | 3.73 | 0.35 | 0.75 | 0.86 | 1.2 |
| --Lachnospiraceae | 2.04 | 2.56 | 0.38 | 0.72 | 0.55 | 0.62 |
| ---*Unclassified*  *Lachnospiraceae* | 1.31 | 1.65 | 0.22 | 0.44 | 0.39 | 0.46 |
| -Bacilli | 6.86 | 7.21 | 35.2 | 28.65 | 15.09 | 12.92 |
| ---*Bacillus* | 0.37 | 0.44 | 1.2 | 1.64 | 1.2 | 1.25 |
| --Staphylococcaceae | 0.63 | 0.95 | 7.05 | 10.54 | 4 | 4.6 |
| ---*Staphylococcus* | 0.41 | 0.56 | 5.38 | 9.86 | 2.34 | 3.63 |
| ---*Salinicoccus* | 0.14 | 0.55 | 1.06 | 1.33 | 1 | 0.89 |
| --Streptococcaceae | 0.6 | 0.81 | 17.82 | 33 | 0.53 | 0.8 |
| ---*Streptococcus* | 0.6 | 0.81 | 17.82 | 33 | 0.53 | 0.8 |
| --Planococcaceae | 0.39 | 0.47 | 0.91 | 1.08 | 1.73 | 1.78 |
| --Bacillaceae | 1.62 | 3.65 | 6.19 | 9.9 | 5.76 | 9.89 |
| ---*Unclassified*  *Bacillaceae* | 0.36 | 0.89 | 2.92 | 7.03 | 2.15 | 5.23 |
| ---*Unclassified*  *Bacillaceae* | 0.88 | 2.72 | 2.07 | 3.04 | 2.41 | 4.04 |
| --Aerococcaceae | 0.22 | 0.36 | 1.33 | 1.3 | 1.06 | 1.03 |
| --Gemellaceae | 2.52 | 4.15 | 0.4 | 0.52 | 0.52 | 0.84 |
| ---*Unclassified*  *Gemellaceae* | 2.52 | 4.25 | 0.5 | 0.52 | 0.52 | 0.84 |
| **Proteobacteria** | 52.57 | 27.36 | 30.66 | 22.26 | 42.18 | 22.2 |
| -Gammaproteobacteria | 40.17 | 32.82 | 22.85 | 22.97 | 28.58 | 26.16 |
| --Unclassified  Cardiobacteriales | 6.56 | 11.42 | 2.67 | 10.33 | 12.08 | 30.75 |
| ---*Unclassified*  *Cardiobacteriales* | 6.56 | 11.42 | 2.67 | 10.33 | 12.08 | 30.75 |
| --Pseudomonadaceae | 1.16 | 0.95 | 1.21 | 1.21 | 1.48 | 1.55 |
| ---*Pseudomonas* | 1.05 | 0.82 | 0.99 | 1.05 | 1.34 | 1.56 |
| --Pasteurellaceae | 17.73 | 31.53 | 3.1 | 9.72 | 3.76 | 6.18 |
| ---*Unclassified*  *Pasteurellaceae* | 17.41 | 31.52 | 3.01 | 9.74 | 3.52 | 6.26 |
| --Enterobacteriaceae | 1.48 | 1.73 | 1.14 | 2.36 | 1.09 | 2.18 |
| --Moraxellaceae | 11.64 | 23.14 | 11.73 | 22.13 | 7.52 | 8.45 |
| ---*Moraxella* | 8.78 | 23.03 | 0.75 | 1.84 | 0.87 | 1.84 |
| ---*Acinetobacter* | 1.8 | 2.79 | 10.5 | 22.16 | 5.57 | 8.61 |
| --Xanthomonadaceae | 0.56 | 0.78 | 1.55 | 1.57 | 1.25 | 1.04 |
| -Alphaproteobacteria | 8.08 | 6.05 | 5.63 | 5.11 | 8.64 | 6.36 |
| --Sphingomonadaceae | 3.8 | 2.99 | 1.42 | 1.29 | 2.56 | 2.21 |
| ---*Sphingomonas* | 3.06 | 2.71 | 0.54 | 0.64 | 1.34 | 1.22 |
| -Betaproteobacteria | 3.56 | 8.37 | 1.55 | 1.23 | 4.18 | 7.61 |
| --Neisseriaceae | 2.34 | 8.18 | 0.11 | 0.17 | 2.17 | 7.7 |
| **Bacteroidetes** | 6.94 | 5.52 | 4 | 2.93 | 5.42 | 4.26 |
| -Bacteroidia | 4.31 | 4.86 | 0.79 | 1.09 | 1.23 | 1.61 |
| --Unclassified  Bacteroidales | 2.39 | 3 | 0.28 | 0.57 | 0.55 | 0.91 |
| ---*Unclassified*  *Bacteroidales* | 2.39 | 3 | .28 | 0.57 | 0.55 | 0.91 |
| **Actinobacteria** | 19.1 | 19.54 | 24.42 | 16.97 | 29.70 | 18.43 |
| -Actinobacteria | 18.11 | 19.65 | 23.1 | 16.58 | 27.53 | 18.55 |
| --Nocardioidaceae | 0.67 | 0.56 | 1.07 | 0.83 | 1.16 | 1.07 |
| --Micrococcaceae | 1.48 | 1.83 | 2.62 | 2.19 | 2.91 | 2.65 |
| ---*Arthrobacter* | 0.86 | 1.82 | 1.16 | 0.93 | 1.82 | 2.1 |
| --Dermabacteraceae | 0.08 | 0.16 | 1.04 | 1.13 | 1.36 | 1.28 |
| ---*Brachybacterium* | 0.08 | 0.16 | 1.04 | 1.13 | 1.32 | 1.23 |
| --Intrasporangiaceae | 0.3 | 0.44 | 2.28 | 2.64 | 2.16 | 2.61 |
| ---*Unclassified*  *Intrasporangiaceae* | 0.16 | 0.39 | 0.39 | 0.65 | 0.46 | 0.68 |
| --Corynebacteriaceae | 6.47 | 16.92 | 10.1 | 11.88 | 11.28 | 19.92 |
| ---*Corynebacterium* | 6.47 | 16.92 | 10.1 | 11.88 | 11.28 | 19.92 |
| --Gordoniaceae | 4.78 | 12.07 | 1.27 | 1.64 | 1.15 | 1.65 |
| ---*Gordonia* | 4.78 | 12.07 | 1.27 | 1.64 | 1.15 | 1.65 |
| --Unclassified  Actinomycetales | 0.66 | 0.8 | 0.48 | 0.59 | 1.07 | 0.89 |
| ---*Unclassified*  *Actinomycetales* | 0.66 | 0.8 | 0.48 | 0.59 | 1.07 | 0.89 |
| --Microbacteriaceae | 1.27 | 1.36 | 0.61 | 0.66 | 1.2 | 1.25 |
| **Verrucomicrobia** | 1.58 | 1.78 | 0.57 | 0.73 | 0.64 | 1.07 |
| -Verruco-5 | 1.44 | 1.76 | 0.34 | 0.68 | 0.4 | 0.91 |
| --RFP12 | 1.44 | 1.76 | 0.34 | 0.68 | 0.4 | 0.91 |
| ---*Unclassified RFP12* | 1.44 | 1.76 | 0.34 | 0.68 | 0.4 | 0.91 |
