# Supplementary figures and images for: Alterations of the bacterial ocular surface microbiome are found in both eyes of horses with unilateral ulcerative keratitis
Source: PLoS One. 2023 Sep 8;18(9):e0291028. doi: 10.1371/journal.pone.0291028 (PMC10490969; doi:10.1371/journal.pone.0291028)

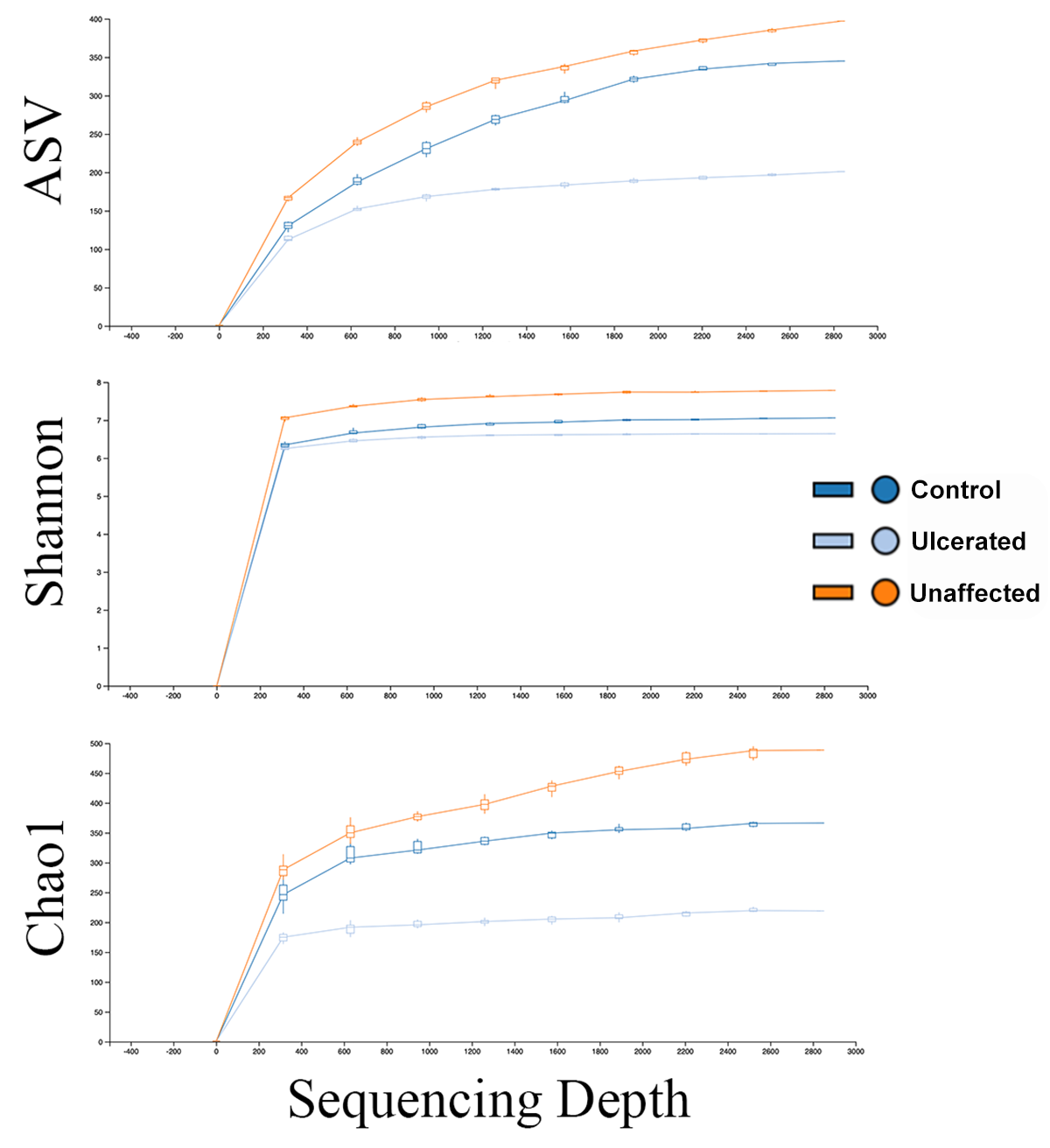

Supplement: S1 Fig — (TIF) [file pone.0291028.s003.tif]

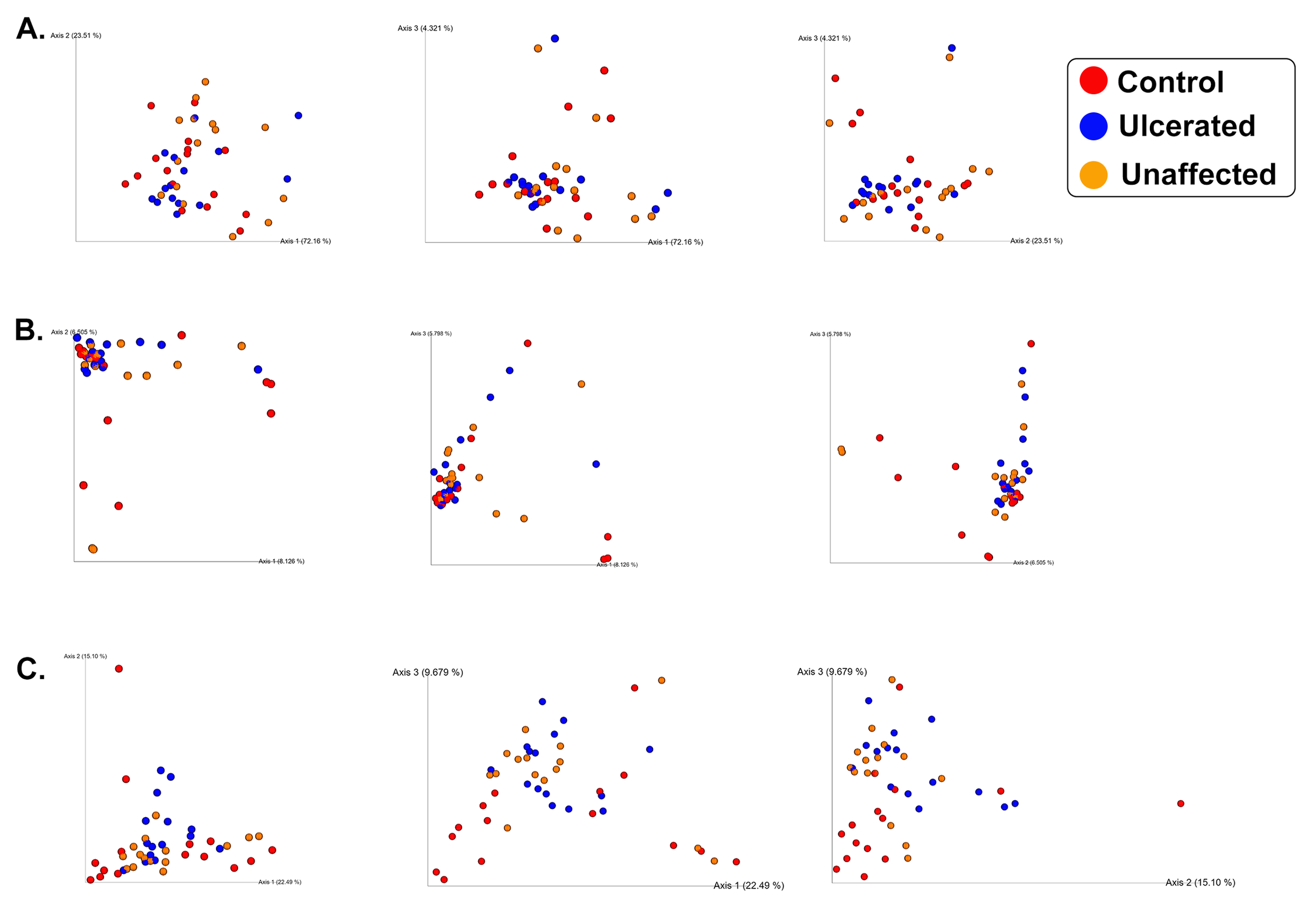

Supplement: S2 Fig — Principal coordinate analysis (PCoA) 2D plots of (A) Aitchison, (B) Bray-Curtis, and (C) weighted UniFrac distance matrices between control (red), ulcerated (blue) and unaffected (orange) eyes of horses. Each dot represents the microbial composition of one eye. Clustering was observed indicating a significant difference in community structure and relative abundance of taxa detected between ulcerated eyes of horses with UK and healthy control eyes (Bray Curtis: R = 0.205, p = 0.002; weighted UniFrac: R = 0.183, p = 0.001). The remaining distance matrices and comparisons showed no difference in community structure (Aitchison: R = 0.03 control vs. unaffected, R = 0.019 control vs. ulcerated, R = 0.039 ulcerated vs. unaffected; Bray-Curtis: R = 0.064 control vs. unaffected, R = -0.011 ulcerated vs. unaffected; weighted UniFrac: R = 0.05 control vs. unaffected, R = 0.018 ulcerated vs. unaffected; p > 0.05). (TIF) [file pone.0291028.s004.tif]
